# Physiology and Legislation
**Essais Physiologiques sur la Législation.*—Premier Essai:—De l'Interdiction des Aliénés. Par H. De Castelnau, Rédacteur-en-Chef du *Moniteur des Hôpitaux et des Sciences Médicales,* ancien Inspecteur-Général adjoint des Prisons et des Etablissements d'Aliénés de France, &c. 8vo. Pp. 202. Paris, 1860.


**Published:** 1861-04

**Authors:** 


### ART. XII.—PHYSIOLOGY AND LEGISLATION.*

THE philosophy of law is a subject which must always possess attraction for thoughtful and educated minds. Its scope is dependent on the development of the human intellect, and as this progresses, new laws are brought into view and new applications of principles, the evolving and orderly arrangement of which will at all times be to the student at once the object and the reward of his labour. As, however, art precedes science, the jurist is not in general the legislator. Legislation is a tentative process; laws are the immediate offspring of experience, and the great practical use of jurisprudence is not exhibited in originating laws, but in correcting, moulding, and harmonizing them. The recognised course of legislation is "to remedy experienced evils and to extend experienced benefits," and the speculative reformer has a long contest before him who would advocate changes by way of conclusion deduced from theoretical considerations.

The author of the work before us is of opinion that,—

"The philosophy of law at the present day is scarcely to the true science of legislation what alchemy was to the science of Lavoisier and Berzelius." (p. x.) "That philosophical legislators hitherto have possessed but a Platonic love of nature, and have erred in thinking they could interpret and understand it without observation; that they could, by the mere reflection of the consciousness upon itself, by the unaided effort of reason, know the entire man without studying his organization and his functions, or the organization and

---

* *Essais Physiologiques sur la Législation.*—Premier Essai:—De l'Interdiction des Aliénés. Par H. De Castelnau, Rédacteur-en-Chef du *Moniteur des Hôpitaux et des Sciences Médicales*, ancien Inspecteur-Général adjoint des Prisons et des Etablissements d'Aliénés de France, &c. 8vo. Pp. 202. Paris, 1860.

functions of other organized beings; that it was, in fact, possible to regulate his actions, that is to say, to formulate a theory for this object without bringing to the consideration of the various psychological phenomena which essentially constitute them, the certitude which is required as to all other natural phenomena not only before attempting their systemization, but even before considering them as definitively acquired by the particular science to which they relate." (p. xi.) "The true science of man, that is to say, physiology understood in its widest acceptation and in its highest sense, is the indispensable torch of every one who would aspire to give laws to his equals, for these laws, though apparently all conventional, ought not the less to be derived, in order to be just, and, probably, in order to be durable, from natural laws; but how can we draw conclusions if we are ignorant of the premises, and where are the premises, that is to say, the natural laws of man, if not in physiology?" (p. 4.)

The present work is an application of these views to the special subject of the French law relating to the "interdiction" of the insane. The application for the interdiction of a lunatic according to the French law is equivalent to our commission in lunacy; but the former has the advantage of being much simpler, and, consequently, less expensive. M. Castelnau mentions incidentally, that judgment of interdiction is obtained annually in upwards of six hundred cases, whereas we learn from the "Judicial Statistics" of this country for 1859, that the number of "Orders of Inquiry" in lunacy executed in that year was only sixty-nine, which is probably rather more than the number of lunatics whose estates and persons were committed to legal control under the authority of the Great Seal in the same year. The expense of this process, which the commissioners report is never less than 75*l.*, doubtless accounts in a great measure for the difference in the numbers of persons put under legal control in the two countries, and also confirms the commissioners' statement as to the necessity for the amendment and simplification of our own process.

The basis of the law of interdiction is contained in the 489th Article of the *Code Civil,* which provides that "An adult who is habitually in a state of idiocy, dementia, or furor, may be interdicted even though such state present some lucid intervals." The appointment of a guardian for the lunatic follows upon the judgment of interdiction; and Article 509 declares that "A person interdicted bears likeness to a minor as regards his person and his property; and the laws concerning the guardianship of minors shall be applicable to the guardianship of persons under interdiction." Article 450, Tit. x., *Of Minority, Guardianship, &c.*, provides "that the guardian shall have the care of the person of the minor, and shall represent him in all civil acts. He shall deal with his property like a good father of a family." Such is an outline of the law to which our author seeks to apply the touchstone of physiology. Its weak point is in the words describing the class subject to interdiction, in Article 489. This Article suggested a memoir which M. Brierre de Boismont read about thirty years ago before the Academy of Sciences:

"He could not," he informs us, "from his own observation and experience, do otherwise than perceive its extremely limited application, inasmuch as cases of furor may have been common when patients were chained, beaten, and exhibited like wild beasts; but this state of things no longer exists; and in well-conducted establishments we seldom witness states of furor excepting as a temporary (*passager*) symptom of acute mania. The legal signification of the word dementia is very different from that which physicians understand by it as indicating a state of chronic debility, and sometimes rapid failure of the intellect. A very considerable class of insane persons—monomaniacs—are not even mentioned. Whatever latitude may be given to this 489th Article, logically and practically speaking, it would be impossible to include under either of these three denominations that singular aberration of mind which dwells on one idea, or rather on a series of ideas, while the person so affected appears to preserve the integrity of his reason on all other subjects."*

In its legal construction, we learn from M. Castelnau that the article in question applies to those "who cannot direct their conduct according to the dictates of ordinary reason, who are unable to manage their affairs, or are incapable of consecutive reasoning, and those who yield without resistance to all their desires; in a word, to those who, according to an expression frequently employed, but never understood, are not in the enjoyment of their *free will*." (p. 28.)

"It concerns us," says M. Castelnau, "to decide whether the physical reform wrought by Pinel may not now be completed by a moral reform; whether, in the same way that the illustrious alienist succeeded in delivering without danger a great number of unhappy beings from the bad treatment inflicted on them, causing them to breathe a purer air, and leaving them the free exercise of their limbs, we may not, without any increase of inconvenience, restore the greater number of them to moral life, and preserve them the enjoyment of liberty as the first of the blessings which civilized society ought to guarantee to man.

"Liberty is wrested annually, in France, by the application of Article 489 of the *Code Civil*, from more than six hundred citizens, guilty solely of having undergone an alteration, more or less marked, of the intellectual faculties, and of possessing property; and not only do they lose the liberty, in some sort physical and savage, of directing

* See *Journal of Psychological Medicine*, vol. v. p. 466.

their steps whither their will leads them, of satisfying their appetites when they feel the wish; but also that moral liberty born of civilization, more precious still than the first, of disposing of their goods either during life or after death, of dispoing even of their person, and of seeking in the pure consolations of marriage and paternity an alleviation of their ills." (p. 9.)

Before proceeding to criticise the reasons and facts of the compilers of the *Code Civil*, M. Castelnau takes occasion to recal a principle, the forgetfulness of which on their part he deems has caused them to stray from the path of justice; and this principle is that of individual liberty, " which itself leads us to the foundations of all society." The consideration of this brings us to the first attempt to construct a law upon the basis of physiology.

"Theologians, and unfortunately, also, nearly all philosophers," writes our author, "have accumulated hypotheses, reasonings, volumes, and errors in order to fix these foundations on immovable bases; they have limited some to narrow, sometimes cruel doctrines, in opposition to the most evident laws of Nature; at which reason, if not both reason and humanity, consequently revolt; others to systems sometimes ingenious, sometimes *bizarre* or even ridiculous, and almost always impracticable, and whose least defect sometimes is, that they are wholly unintelligible. Is our destiny, then, an insoluble problem, or have our philosophers rather been wanting in genius? Neither the one nor the other: the destiny of man appears to us to be written in his organization, and philosophers have but wanted science and method. Without committing myself to demonstrations unsuited both to time and place, it will suffice for the object I wish to attain to recal,

" 1st. That of all laws of organization, the most imperious is that which leads us to seek after pleasure and to fly from pain.

" 2nd. That the instinct of sociability is not only an irresistible need of our nature, but a necessity imposed upon our individual weakness.

" 3rd. That the satisfaction of these two needs requires that society respects our life, our property, and our liberty, and that we respect the life, property, and liberty of our fellow-men.

" These are primary truths, susceptible, nevertheless, of demonstration, but which, in consideration of their being obvious, we may be permitted to consider as axioms of sociology It results, then, from these axioms that, in a society whose laws are conformable to those of nature, the liberty of a citizen would have no limits beyond those imposed by the liberty of others.

" Whoever, then, does not endanger the liberty of others ought to live free in society, whether he be, as we believe, a wise man or a madman: in respect to the right to liberty there is no difference between them." (p. 22.)

This appears to be a very important part of M. Castelnau's argument, and we have given it in his own words; but the first proposition involves a sophism so often and so long ago exposed, that it is with a feeling akin to astonishment we see it intruded again into a serious argument. Plato has put certain words into the mouth of Socrates, in the *Gorgias*,* which, from their apt bearing upon the point in question, we may be forgiven for quoting at large:—

"*Soc.* Now listen to me while I recapitulate the discourse which we have had from the beginning. The good and the pleasant are not identical, as I and Callicles agreed. Is the good to be sought for the sake of the pleasant or the pleasant for the sake of the good? The pleasant for the sake of the good. Now *that* is pleasant by the presence of which we receive pleasure; *that* is good by the presence of which we are good. But we are good, and everything else which is good is good by the presence of some goodness or virtue. But the goodness or virtue of anything, whether it be a body or any bodily structure, or a living thing and a soul, cannot be a thing which belongs to it by chance and accident; it must come by some order and appropriateness and rightness of its parts. And so virtue in everything is something implying a certain order in its parts—a constitution. In every kind of thing its appropriate constitution it is which makes it good. And thus a soul which has its proper constitution is better than one which has not. Such a soul is rightly constituted. But a rightly-constituted soul is under control, is temperate. And so a soul which is temperate is good, a soul which is intemperate is bad. And a temperate soul under due control will do what is right towards the gods and towards men. It would not be under due control if it did not. Now what is right towards man is *justice;* what is right towards the gods is *piety:* and he who does such things is *just* and *pious.* And such a man will also be *brave;* for he whose soul is under due control will seek what he ought to seek and fly what he ought to fly, be it acts or men, or pleasures or pains, and will endure the stress of pain or danger when he ought to do so. And thus the man of rightly-regulated soul being, as we have said, just, and brave, and pious, must be in all respects a good man, and, as a good man, must do well, whatever he does. And he who does well must be happy, and the bad man, who does ill, must be wretched. And this bad man must be the man who is under no control. . . . And if it be true, I say that he who desires to be happy must aim at and practise self-control, and must shun the absence of control with all his powers. He must endeavour, in the first place, not to need correction; but if he need it, or if any one that he cares for does, man or state, he must try that it may be bestowed, if there is to be any hope of happiness. This seems to be the purpose, end, and aim of life. We must try as much as possible to cultivate temperance and justice if we wish for happiness. We must not, as has been held, let our desires expand uncontrolled, and then try to fill them—a bottomless abyss of evil—the life of a highway robber. A man who takes that course must be hateful to men and gods. He can have no fellow-feeling with other men, and when there is no fellow-

* Whewell's *Platonic Dialogues*, vol. ii. p. 223.

feeling there can be no friendship. And the wise affirm, Callicles, that heaven and earth, and gods and men, are held together by the attraction of a general sympathy—by friendship, and orderliness, and control, not by disorderliness and uncontrol."

Unless we are prepared to say that the Good and the Pleasant are identical, how can we admit the proposition, that the most imperious law of our organization is that which leads us to seek after pleasure? And if we believe that control in some shape is necessary to the happiness of all men, how shall we say that the man who is incapable of self-control, "who yields without resistance to all his desires," is not to be controlled? Surely, if we have any regard for him, we must endeavour, for his own sake, not less than with a view to our own interest, to supply his deficiency. Again, if control is universally necessary to happiness, the man incapable of self-control must be happier under the wise control of others than he would be if uncontrolled, and we are therefore in no way bound to admit with M. Castelnau, except in a much wider sense than would suit his argument, that—

"To act in the interest of the insane, in interdicting him, can only be understood to mean one of the following things:—1st, To soothe his sufferings; to restore his health, or at least to hinder him from hurting it, by the acts of a disorderly life. 2nd, To assure the satisfaction of his legitimate desires, and to preserve him his property and the full enjoyment of his income."

This proposition is made as the basis of an argument, the irrelevancy of which is sufficiently apparent. "Health," says our author, "is undoubtedly a great blessing, for without it life is but a long martyrdom, a prolonged vegetation; but there is a greater blessing," and, quoting a saying of "the judicious Procureur-Général Merlin," he says, "Liberty is the greatest of blessings, that of which man shows himself most jealous." Further, he concludes that "if, in order to give the first, one is obliged to take away the second, one takes away the greater to give the less." But absence of control does not constitute liberty, neither is the Procureur's panegyric applicable to licence.

But M. Castelnau attempts to prove by statistics that the judgment of interdiction exercises a prejudicial influence on the persons subject to it, and is chargeable with lessening the number of cures amongst them, as compared with the cures amongst the general class of insane patients who are not interdicted. We are bound to say that, to our apprehension, the proof is wanting. The following are his statements upon this point:—

"The science of mental therapeutics at the present day pretends to cure one patient out of three. This pretension does not appear to me to be established by data free from all objection, but everything leads to the conviction that, if not rigorously true, it approximates to the truth very closely. The latest statistics give, for the year 1853, one cure out of 3·27, or 30·5 per cent; and although these statistics do not furnish all the materials necessary, the figures that they give yield an approximation sufficiently close to enable us to neglect, without inconvenience, the possible error. One cure out of 3·27 is not a result of which science has any reason to be proud, and it is not for such a prospect that one would desire to interdict a patient, if one had regard solely to the interest of his health. What should we say, then, if, by a mystery at present impenetrable, the interdiction reduced by SEVEN OR EIGHT TIMES this chance of cure, and if, instead of having, like the generality of patients, one chance of cure out of three and a quarter, the patient, from the time he is interdicted, should have but one out of TWENTY-THREE AND A THIRD? Such is nevertheless the frightful reality. This is how it is arrived at:—

"*Interdiction ceases with the causes which have determined it.* Such is the law. The malady constitutes the causes. When a cure is obtained, the causes no longer exist; the interdiction ought therefore to be taken off. The number of cures may then be rigorously estimated by the number of judgments of *main-levée* of interdiction.

"During six years, then, taken at hazard (1842, 1843, 1844, 1847 1848, 1857) there were 3201 interdictions pronounced, and 137 judgments of *main-levée* of interdiction; equal to one cure out of 23·38." (p. 43.)

Nothing can be more fallacious than statistics when appealed to after this fashion. No information is given as to the ages of the two classes of patients, the average duration of the disease in either class, or the specific character of the disease. It is quite possible that many persons who have been interdicted may be discharged cured, who do not apply for the judgment of *main-levée*, but, probably, the most important source of error would be found in the different meanings of the word "cured," as applied to the two classes of patients. It is exceedingly probable that a patient would be discharged from an asylum as cured, under circumstances in which it would be difficult for a court of justice to recognise his competency. It must be remembered that, once a man is declared insane, the burden of proof is upon him when he applies to be discharged. Lord Eldon one time observed, "that there was no part of the jurisdiction in lunacy more unpleasant, and requiring greater caution, than that of determining when a commission should be superseded; for, though a safe conclusion may, upon evidence, be arrived at in establishing lunacy, it is very difficult to determine when the mind has been restored." But, after all, M. Castelnau's whole argument is directed against the results of interdiction, viz. the loss of liberty, of the right to dispose of property, and of the right to marry. Now, are not all lunatics under treatment subject to these disabilities? and, if so, there is no difference, as far as we know, between the treatment of the interdicted and the non-interdicted beyond the mere judgment of interdiction pronounced by the Court; and statistics which show that the effect of this judgment is so disproportionate as M. Castelnau suggests, really prove too much. If M. Castelnau's argument is good, and if the greater number of those against whom formal judgment of interdiction is pronounced, are improperly deprived of personal liberty, and of the right to dispose of property, and to marry, *à fortiori*, we should think a much greater number of those under treatment, not being interdicted, must be improperly deprived of those rights. But the medical treatment of lunacy is so dependent upon the power of control over the patient, which involves the suspension of these rights, that, without it, all treatment according to our present knowledge must be abandoned, and yet, as M. Castelnau admits, we cure one patient out of three. Surely, this is a result of too great value to be given up on mere theoretical grounds. Few will be disposed to question that all forms and shapes of insanity do not call for the same degree of direct control; as, for instance, there are many persons undoubtedly insane in the strictest sense of the word, whom it is not necessary to confine in an asylum, and who ought not to be so confined, provided they are so circumstanced as to admit of their having the necessary degree of control and supervision combined with a greater amount of liberty than is compatible with the discipline of an asylum. But we deem it logically impossible to concede to any person formally adjudged to be habitually in a state of idiocy, dementia, or furor, absolute liberty of his person, or the like liberty to dispose of his property. With respect to the right to marry, M. Castelnau argues as though he thought the law debarring a lunatic of this right, depended upon the inheritability of insanity, whereas it follows, from his general incompetence to bind himself by any contract, and if not the necessary result of his incompetency to dispose of his property, depends upon the same arguments. There seems abundant reason, from what M. Castelnau says, to conclude that there is much need for reform, either in the law or the administration of the law, with reference to the property of lunatics. We may say the same at home. The subject has been found very difficult to deal with, but hitherto there has been no doubt with us about the principle, that a lunatic so found by inquisition is properly disqualified for absolute liberty of person, or the usual rights of property. There is, perhaps, less room for any such doubt with respect to persons found lunatic by inquisition, than with respect to persons who may be properly interdicted under the law as it stands in France. The careful accuracy of our legal forms in reference to this subject is worthy of notice, and contrasts favourably with the looser expressions of the French code. Thus, before the Lord Chancellor can commit the custody of the person and property of a lunatic to a guardian, it must be found that the lunatic "is of unsound mind, *and incapable of managing himself or his affairs*." Even M. Castelnau could scarcely object to the conclusion based upon such data.

We cannot terminate our notice of M. Castelnau's work without, in some measure, qualifying some of the preceding remarks by a general expression of satisfaction at the spirit of the essay. The writer's object is undoubtedly one in which all our readers will wish him success; and, in the treatment of his subject, he brings together the ideas respecting insanity held by the compilers of the Code and upon which its provisions are based, and he criticises them with much discrimination. Now that we are about to enter on the discussion of a large measure of reform, it cannot be amiss to consider what our neighbours have done, and are doing, on the same subject, and there is much in the present volume deserving of careful consideration.

---

